# Feasibility and tolerability of moderate intensity regular physical exercise as treatment for core symptoms of attention deficit hyperactivity disorder: a randomized pilot study

**DOI:** 10.3389/fspor.2023.1133256

**Published:** 2023-05-15

**Authors:** L. A. Svedell, K. L. Holmqvist, M. A. Lindvall, Y. Cao, M. Msghina

**Affiliations:** ^1^ Department of Psychiatry, Faculty of Medicine and Health, Örebro University, Örebro, Sweden; ^2^ Department of Neurology and Rehabilitation Medicine, Faculty of Medicine and Health, Örebro University, Örebro, Sweden; ^3^ University Health Care Research Center, Faculty of Medicine and Health, Örebro University, Örebro, Sweden; ^4^ Clinical Epidemiology and Biostatistics, School of Medical Sciences, Faculty of Medicine and Health, Örebro University, Örebro, Sweden; ^5^ Unit of Integrative Epidemiology, Institute of Environmental Medicine, Karolinska Institutet, Stockholm, Sweden; ^6^ Department of Clinical Neuroscience, Karolinska Institutet, Stockholm, Sweden

**Keywords:** ADHD, physical exercise, randomized controlled trial, cognition, impulsivity, hyperactivity, emotion regulation

## Abstract

**Background:**

Attention deficit hyperactivity disorder (ADHD) is associated with sedentary lifestyle, low quality of life and low physical fitness. Studies in children with ADHD have shown that regular physical exercise can help reduce core ADHD symptoms, but evidence for this is lacking in adults. Although guidelines recommend multi-modal treatment, central stimulants (CS) remain the mainstay of treatment. CS are effective in the short-term, but their long-term efficacy remains to be established. There is thus huge unmet need for developing non-pharmacological treatment options, and for well-designed randomized controlled trials (RCTs).

**Objective:**

The study aimed to test the feasibility and tolerability of structured moderate-intensity 12-week physical exercise program for adults with ADHD, as a prelude to an adequately powered RCT which includes long-term follow-up.

**Materials and methods:**

Fourteen adults with ADHD were recruited, 9 randomized to an intervention group and 5 to a control group. The intervention group received physiotherapist-led 50-minute mixed exercise program, three times a week for 12 weeks, and the control group treatment as usual. Participants were assessed at baseline and after 6 and 12 weeks using clinical and physical evaluations, self-rating questionnaires, and functional magnetic resonance imaging (fMRI) together with paradigms that tested attention, impulsivity and emotion regulation.

**Results:**

Three participants (21%) dropped out shortly after inclusion before receiving any intervention, while roughly 80% completed the intervention according to protocol. One participant from the intervention group participated in less than 60% of treatment sessions, and one who had done baseline fMRI was unwilling to do post-intervention imaging. Four participants in the intervention group (67%) reported increased stress in prioritizing the intervention due to time-management difficulties. Overall, consistent trends were observed that indicated the feasibility and potential benefits of the intervention on core ADHD symptoms, quality of life, body awareness, sleep and cognitive functioning.

**Conclusion:**

Physiotherapist-led twelve-week regular physical exercise is a feasible and potentially beneficial intervention for adults with ADHD. There was a 20% drop-out initially and 67% of those who completed the intervention reported stress with time management difficulties due to participation. A third arm was thus added to the planned RCT where cognitive intervention administered by an occupational therapist will be given together with physical exercise.

**Clinical Trial Registration:** https://clinicaltrials.gov, identifier NCT05049239.

## 1. Introduction

Roughly 3% of the adult population is believed to have attention deficit hyperactivity disorder (ADHD) (1–5). Patients with ADHD are more likely to underperform at school or work and run a higher risk of being unemployed or on long-term sick leave (1, 2). Although guidelines recommend multimodal treatment with a combination of pharmacological and non-pharmacological interventions (6), central stimulants still remain the mainstay of treatment (7). Central stimulants are highly effective in reducing symptom and improving function, but evidence for their long-term efficacy and safety in an aging adult ADHD population requires further investigation (7, 8). The majority of patients respond well to pharmacological treatment with stimulant medication, but approximately 30% respond poorly or discontinue treatment because of side effects even when treatment is found to be effective in reducing symptoms (9). There is thus a huge unmet need for developing non-pharmacological treatment options (10, 11), and for well-designed randomized controlled trials (RCTs) to test this.

Adults with ADHD often have sedentary lifestyle (12), sleep disturbance (18), obesity (13, 14), low quality of life and comorbidities such as depression and anxiety disorders (11). In the general population, physical exercise has been shown to mitigate the negative impact of these known risk factors for physical and mental illness (15). Besides the general health benefit in mitigating the known risk factors that are overrepresented in ADHD patients, physical exercise may also have a specific positive effects on core symptoms of ADHD and thus needs to be further investigated as a potential specific treatment option for adults with ADHD. Studies in children and adolescents with ADHD have shown that physical exercise may improve core symptoms of ADHD and cognitive functioning (16–24), but evidence for this is lacking in adults (21, 25). Single session physical exercise lasting >20 min has been evaluated in a number of studies and reported to have acute beneficial effects on some measures of cognitive functioning in children with ADHD (26, 27). Cumulative effect of regular physical exercise with multiple sessions over a longer period has also been evaluated in children with ADHD. Ahmed and Mohamed (2011) studied a 10-week, three times a week 50 min aerobic exercise and found positive effects on core symptoms in children with ADHD (28). Kang et al. (2011) in a similar 6-week, three times a week aerobic exercise at 60% of maximal heart rate also found positive effects of regular physical exercise in children with ADHD (29). A resting-state functional magnetic resonance imaging (fMRI) study in children with ADHD found that 8-week rope-skipping aerobic exercise improved executive function, with concomitant changes in spontaneous activations in the left middle and right superior frontal gyri (30). A recent meta-analysis evaluating the efficacy of different types of physical exercise in children and adolescents with ADHD found a mixed exercise program to be the most promising in reducing symptoms (31). Another meta-analysis (32) that included 15 RCTs in children with ADHD found that regular physical exercise improved attention and executive function, but not hyperactivity or aggressive behavior. Based on these findings in children and adolescents, we hypothesized that a physiotherapist-led moderate intensity mixed exercise program could help adults with ADHD in improving core symptoms and in reducing risk factors for other physical and mental co-morbidities. We thus plan to conduct an adequately powered 12-week randomized controlled study to investigate the effects of regular physical exercise on core symptoms of ADHD and measures of cognitive and affective functioning, with evaluations directly at the end of the intervention and at 6 and 12 months after the start of the intervention. The primary aim of this pilot study was to test the feasibility and tolerability of the intervention, gain experience and collect data that will help optimize the planned RCT. A secondary objective was to assess any preliminary trends in improvement in ADHD symptoms and/or measures of general health and daily functioning.

## 2. Materials and methods

### 2.1. Study design

The present study was a randomized controlled pilot trial in which the intervention group received a 50 min structured mixed physical exercise program three times a week lasting 12 weeks, and the control group received treatment as usual (TAU). Participants in both the intervention and control groups were allowed to have any ongoing ADHD treatment other than regular physical exercise. However, pharmacological treatment had to be stable for two weeks prior to inclusion and no major change in this was allowed during the intervention period. The intervention was given at the Unit for Psychiatric Physiotherapy, Region Örebro County, Sweden, by a group of licensed physiotherapists. Participants came up to three times a week for the 50 min mixed exercise intervention, with an option of doing sessions on their own if they could attain a minimum of 60% of maximal heart rate (HR) for 10 min or more. The goal was that participants would be physically active for at least 150 min per week at a moderate intensity level. HR was measured both during the clinic-based and home-based sessions to confirm that participants attained a minimum of 60% of maximal HR. HR was captured using a Polar Heart Rate Monitor (Model H10) and assessed from rate-to-rate intervals that were interpolated every second and transferred to a Polar HR App (Polar Electro Oy, Finland). Each participant had a HR monitor connected to the Polar HR App in the participant's own smartphone to enable HR measurement even during the home-based sessions. HR was not a target variable as such but was used to confirm that participants exercised at the level of the pre-set criteria. The age-predicted equation (220 – age) was used to determine maximum HR.

Ethical approval was obtained from the Swedish Ethical Review Authority (EPM, 2020-05641; 2021-02743) and the study was conducted in accordance with the Declaration of Helsinki.

### 2.2. Participants

Inclusion criteria were adults with ADHD who received diagnosis after structured neuropsychiatric evaluation by a dedicated multidisciplinary team consisting of at least one senior consultant in psychiatry and a licensed psychologist, 18–65 years of age, unmedicated or with stable ongoing pharmacological treatment. All subtypes of ADHD were included in this study, as will also be the case in the planned RCT. Exclusion criteria included comorbid diagnosis of moderate to severe unipolar depression, bipolar disorder, psychosis, substance syndrome and suicidal behavior. Participants were recruited by advertisement in the local community and with the help of brochures left at Örebro University Hospital. Those who expressed interest in participating in the study were contacted by telephone and a visit for additional oral and written information was booked. Once informed consent was obtained, participants were randomized into an intervention group or a control group and assessed for baseline measurement of primary and secondary outcome variables by a team of a senior consultant in psychiatry and a licensed physiotherapist. Self-rated and clinician-rated ADHD symptom measures and measures of physical capacity and body awareness were conducted at baseline, at week 6 and within one week after the end of the intervention. Measures of cognitive functioning with or without concomitant fMRI were conducted at baseline and within one week after the end of the intervention.

### 2.3. Intervention

The intervention consisted of physiotherapist-led mixed exercise program with moderate to high intensity cardio, strength and flexibility exercises in small groups of 4–6 participants. The exercise had a target level of 60%–90% of maximum HR and was given for 50 min, three times a week for twelve weeks. The training sessions took place in the gym at the outpatient clinic or outdoors in a nearby park. Each session followed a specific structure (Table 1). The exercise always started with 5–10 min warm-up on a cycle ergometer or with body movements, followed by a cardio workout in three intervals of one-minute and one-minute active rest between intervals, followed by three sets of resistance exercise and cool down and flexibility exercises for 10 min in the end.

**Table 1:** Structure of the mixed exercise program used as intervention in the present study.

Mixed exercise program (50 min)		
Type	Duration	Content
Warm-up	6 min (12%)	Light exercise^a^
Cardio workout	6 min (12%) in intervals^b^	Cycling, running, jumping
Resistance training	23 min (46%) in intervals^c^	Lower body, upper body, core, balance
Flexibility	10 min (20%)	Stretching of large muscle groups
Cool-down	5 min (10%)	Light exercise

^a^ Cycling, cross-trainer, walking, full body movement (low resistance, comfortable pace).

b 3 × 1-minute intervals, with 1-minute active rest between intervals.

c 3 rounds of 5 × 1-minute intervals with 20-second rest between intervals and 1-minute rest between rounds.

The resistance training included five different exercises performed in one-minute intervals in three rounds. The resistance training differed slightly depending on location (indoors or outdoors), but always included an exercise for leg strength (for example squats or lunges), for arms and shoulders (for example shoulder press with bare bell or bench press), for body-core (for example crunches or the plank) and a balance challenge (for example standing on one leg). Participants did as many repetitions as possible in one minute and the load was then adjusted depending on the number of repetitions that participants managed to do. The physiotherapist offered an alternative exercise if for any reason a participant could not perform a given exercise. Since ADHD is known to negatively affect top-down control of goal-related behavior (6, 33), and this can be expected to compromise adherence to the protocol of the study, care was taken to make sure that study participants had alternative training options. If study participants missed a planned session, they could attend another intervention session on another day of the same week or do the planned exercise session at home and monitor and report level of activity.

### 2.4. Sample size determination, randomization, and blinding

Power and sample size calculation were not performed for the pilot study, as a group of 14 participants was judged to be sufficient to test the feasibility and tolerability of the intervention. To gain more experience in the practicality of the intervention, we opted for a 2:1 randomization ratio and 9 patients were thus randomized to the intervention group and 5 to the control group. Due to the nature of the intervention, it was not possible to blind participants during self-assessment with self-rated health questionnaires. Participants were thus instructed to not reveal the group they belonged to in order to maintain the integrity of evaluator blinding during the clinician-rated assessments.

### 2.5. Assessment and outcome measures

The primary outcome measure was ADHD symptom reduction measured with clinician-rated Clinical Global Impression-Severity/Improvement (CGI-S/CGI-I) (34) and patient-assessed Adult ADHD Self-Rating Scale (ASRS v1.1.) (35). Exploratory secondary outcome measures include Patient-rated Global Impression-Improvement (PGI-I) (34), quality of life assessed with EQ-5D-5l (36) and cognitive functioning evaluated using standardized paradigms [AX-CPT, Go/NoGo and emotion regulation tasks (37–39)] with or without concomitant fMRI. Indicators of general health (BMI, heart rate, blood pressure), body awareness, sleep habits, self-efficacy and daily physical activity were also assessed given the hypothesis that regular physical exercise reduces burden of disease and improves general health in adults with ADHD.

The pilot study was neither designed nor adequately powered to evaluate the effects of the intervention. However, to gain experience with and test the practicality of the assessments, participants were evaluated using the instruments of the planned RCT. The participants thus completed the following questionnaires online using the electronic data capture system Smart-Trial: PGI-I, ASRS-v1.1, MADRS-S (40, 41), EQ-5D-5l, Self-Efficacy Scale (GSE-10) (42), Self-Efficacy for Exercise (SEE-SV) (43) and Insomnia Severity Index (44). Participants were also assessed by a clinician with CGI-S and Body Awareness Scale—movement quality and experience (BAS MQ-E) (45). Number of steps were measured with accelerometer (Model Actigraph GT3X+) (46–48) and VO2 with Ekblom-Bak test submaximal cycle ergometry on a mechanically braked Monark cycle ergometer Model 828E (49, 50). Handgrip strength was measured with a hand-held dynamometer JAMAR (51, 52). The Flamingo balance test, a 60-second one-leg stance-balance test, was used to measure static balance and stability in abdominal, pelvic and leg muscles (53). Height, weight and waist circumference were measured with standardized tests (54) and body mass index (BMI) calculated. As planned for the main RCT, participants also performed cognitive tasks (AX-CPT, Go/NoGo and emotion regulation) with or without concomitant functional magnetic resonance imaging (fMRI); the preset target here was that 50% of the participants would perform the fMRI investigations.

### 2.6. Assessment of participant experience

To investigate participants' experience, a study-specific questionnaire with closed response options was used. The questions were answered on an ordinal scale, from 1 to 5, for each question. In addition to answering the questions, participants were asked to freely comment in running text. The study-specific questionnaire consisted of 40 questions divided into four sections regarding (i) the physical tests, (ii) cognitive tests, (iii) fMRI, and (iv) the intervention itself. Each section contained questions about physical and mental exertion during and after performing the tests, and opinions about time allocation. The questions on the intervention included content, time allocation, joyfulness, opinion about the physiotherapist's ability to lead and motivate, own motivation, physical and mental effort, perceptions of the home training, problems with adherence, changes in mental and physical health and perceived improvement in ADHD symptoms. The participants were also asked to write in their own words about perceived factors that hindered or promoted adherence. Instead of answering questions about the intervention, the control group answered a question of whether the tests had contributed to any change in physical activity.

### 2.7. Data analysis

Descriptive analysis was performed, and the results are reported using mean ± standard deviation and range for continuous variables and count and percentage for categorical variables. Although it was not the main aim of the pilot study, the differences in baseline characteristics and post-intervention changes between the intervention and control group were tested using t-test, Mann–Whitney *U*-test or Fisher's exact test whichever was appropriate. All analyses were performed using the statistical software SPSS version 28.0 (IBM Corp., Armonk, NY, USA). The responses in running text from the study-specific questionnaire were analyzed using manifest content analysis with a low degree of abstraction and interpretation (55). The answers were categorized based on positive and negative experience of the test, motivating factors, hinders and perceived outcomes.

## 3. Results

### 3.1. Baseline characteristics of participants

**Figure 1:**
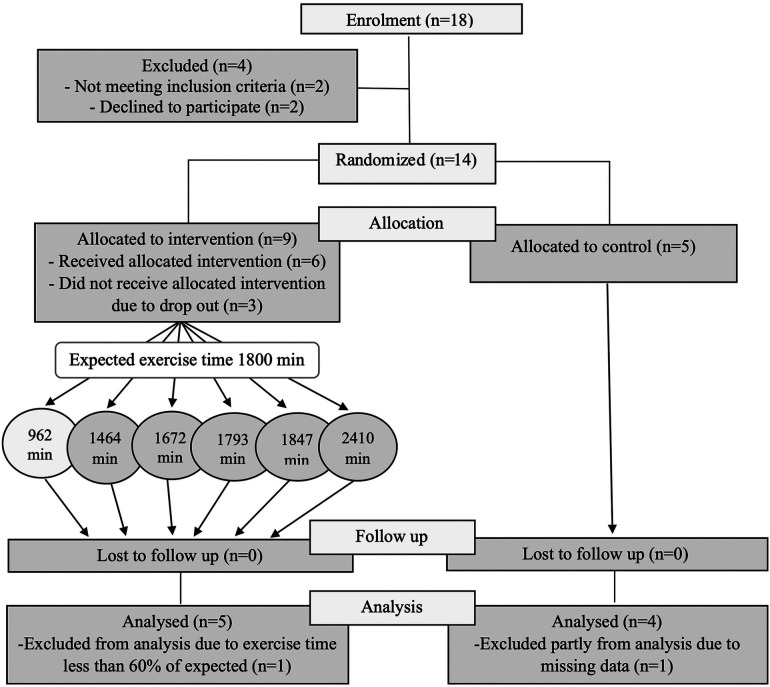
A consort diagram showing the recruitment, allocation, retention and adherence to study protocol.

The catchment area, Örebro county, Sweden, where participants were recruited from, has a population of 300,000, with expected adult ADHD diagnoses of roughly 6,000. Of the 14 participants included in the pilot study, nine were female and five male (Figure 1). Mean age, duration of diagnosis, type and dose of ongoing pharmacological treatment, baseline ASRS v1.1 and Clinical Global Impression—Severity (CGI-S) scores for both the intervention (*n* = 9) and the control groups (*n* = 5) are shown in Table 2.

**Table 2:** Demographic data and baseline value of primary and secondary outcome variables in the intervention and control groups.

Variables	Intervention (*n* = 9)	Control (*n* = 5)	Total (*n* = 14)	Range	*p*-value
Drop-out	3 (33.3%)	0 (0%)	3 (21.4%)		
Age	33.7 ± 8.1	43.0 ± 12.6	37.0 ± 10.7	27–54	0.31
Gender (female/male)	6/3	3/2	9/5		0.53*
Education level					
(High/primary school)	3/6	2/3	5/9		0.74*
Body mass index (BMI)	25.3 ± 5.1	29.1 ± 1.6	27.4 ± 4.6	18.7–33.7	0.15
Moderate risk	2 (22.2%)	3 (60%)	5 (35%)		
High risk ≤30	3 (33.3%)	2 (40%)	5 (35%)		
Waste circumference	92.0 ± 12.7	106.6 ± 1.8	99.3 ± 11.7	73–111	0.15
Moderate risk	2 (22.2%)	0 (0%)	2 (14%)		
High risk	4 (44.4%)	5 (100%)	9 (64%)		
Heart rate (bpm)	87.2 ± 9.3	84.4 ± 20.2	85.8 ± 15.8	57–109	0.84
BP systole (mmHg)	122.4 ± 14.5	134.2 ± 7.0	128.3 ± 12.8	95–140	0.41
BP diastole (mmHg)	78.6 ± 11.9	90.4 ± 7.1	84.5 ± 11.4	75–92	0.42
VO_2_max (ml/kg/min)	2.7 ± 0.6	2.9 ± 0.7	2.79 ± 0.66	1.91–3.85	0.84
Low	4 (44.4%)	2 (22.2%)	6 (67%)		
Very low	1 (11.1%)	2 (22.2%)	3 (33%)		
Duration of diagnosis	5.7 ± 3.3	3.4 ± 1.9	4.64 ± 2.7	1–11	0.22
EQ VAS	50.2 ± 19.6	45.0 ± 17.3	47.6 ± 18.8	25–78	0.75
ASRS V1.1.	40.2 ± 2.3	45.2 ± 8.3	42.7 ± 6.6	34–58	0.42
CGI-S	3.8 ± 1.7	3.8 ± 1.0	3.8 ± 1.4	2–7	0.69
MADRS-S	19 ± 6.2	16.8 ± 7.2	18.2 ± 6.7	6–27	0.55
Medication (yes/no)	5/4	3/2	8/6		0.53*
LDX (mean dose, mg)	60.0 ± 10.0	55.0 ± 5.0	57.5 ± 8.3	50–70	
MPH (mean dose, mg)	47.0 ± 14.4	54.0 ± 0	48.8 ± 12.8	27–60	

Statistical analysis was performed using Mann–Whitney *U*-test with the exception of nominal variables indicated with *where Fisher's exact test was used instead.

### 3.2. Adherence to study protocol

In the intervention group, three out of nine participants dropped out (33%) shortly after randomization before participating in any intervention session, while none dropped out after the start of intervention (Figure 1). There was no drop-out in the control group. The most common reason for dropping out was lack of time, except for one participant who could not attend group sessions due to panic disorder. Thus, 11 of the 14 randomized participants (80%) and all 6 participants who started the intervention (100%) completed the study. Among completers, one participant from the intervention group participated in less than 60% of the intervention sessions (a preset minimum level of participation). And although this participant had roughly similar level of post-intervention changes, the participant was excluded from further analyses. Three participants from the intervention group and two from the control group underwent fMRI investigation before and within one week after the end of intervention. One participant from the intervention group did baseline fMRI but declined to do post-intervention fMRI and was thus excluded from the fMRI analysis.

### 3.3. Adherence to the preset frequency and intensity of the intervention

Of the six participants in the intervention group, five (83%) achieved the preset levels of frequency and intensity of the program for at least 80% of the sessions (Figure 1). As mentioned above, one participant participated in most of the follow-up assessments, but had a participation level of <60% and thus did not attain the preset levels of frequency and intensity of intervention.

### 3.4. Changes in ADHD symptoms and quality of life

**Figure 2:**
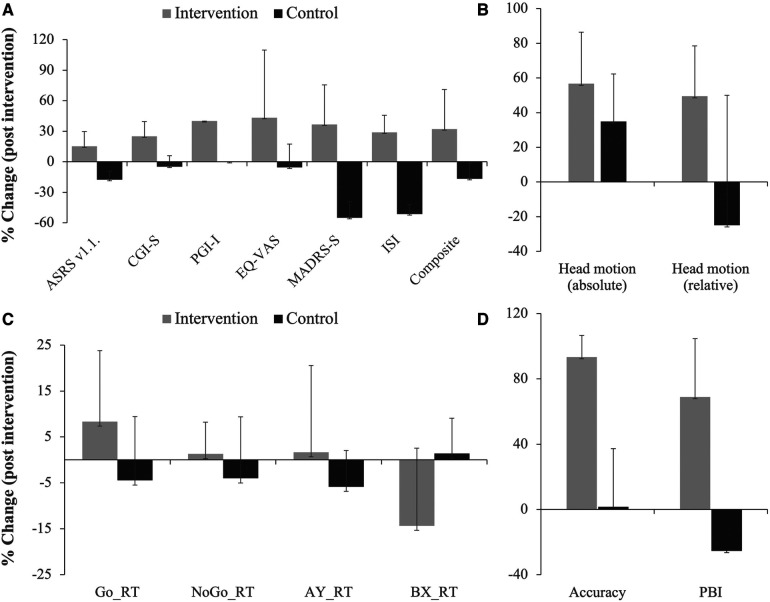
Post-intervention changes in various outcome measures as indicated. Shown are % changes in clinical outcome variables related to symptom (ASRS v1.1.), severity (CGI-S), improvement (PGI-I), quality of life (EQ-VAS), depression (MADRS-S) and sleep disturbance (ISI); included is also a composite score of the first 5 clinical outcome variables (**A**). (**B**) Shows % changes in absolute and relative head motion during fMRI for the intervention and control groups. Results from the cognitive tasks showing % changes in reaction time (RT) during the Go No/Go task and AY and BX trials of the AX-CPT task are shown in (**C**). (**D**) Shows % changes in total accuracy and proactive behavioral index (PBI, calculated from reaction time in the AY and BX trials). Note the consistency in the difference between the two groups in almost all outcome measures. Note even that there was increase in reaction time for the intervention group for the impulsivity measures (Go No/Go and AY trial of the AX-CPT) and decrease in reaction time for the cognitive performance measure (BX trial), while the opposite was the case for the control group.

Post-intervention changes in ADHD symptom rating (ASRS v1.1.), Clinical Global Impression - Severity (CGI-S), Patient-rated Global Impression – Improvement (PGI-I), EQ-5D-5l visual analogue scale (EQ VAS), quality and quantity of sleep assessed with Insomnia Severity Index (ISI), and a composite score calculated for the five clinical outcome variables (ASRS v1.1., CGI-S, PGI-I, MADRS-S and EQ-VAS) are all shown in Figure 2A and Table 3. Although it was not the main aim of the pilot study to test these, many of these outcome variables showed statistically significant improvement in the intervention group but not in the control group (Figure 2 and Table 3).

**Table 3:** Clinical and cognitive outcome variables expressed as post-intervention % change compared to baseline for the intervention and control groups.

Outcome variables	Intervention % change ± SD (range)	Control % change ± SD (range)	*p*-value
ASRS v.1.1.	−15.3 ± 14.4 (−100.0 to +10.0)	+17.6 ± 8.4 (−5.0 to +21.0)	0.036*
CGI-S	−25.0 ± 14.4 (−32.0 to +8.0)	+4.8 ± 10.8 (+2.0 to +21.0)	0.11
PGI-I (responders)	+40	0	0.036*
EQ-VAS	+43.4 ± 66.4(−3.3 to +180.0)	−5.6 ± 23.0 (−55.0 to +7.0)	0.11
MADRS-S	−36.7 ± 38.9 (−100.0 to +9.5)	+55.2 ± 15.9 (−11.5 to +25.0)	0.25
Composite (improvement)^a^	+32.1 ± 11.7 (+5.8 to +54.6)	−16.6 ± 20.1 (−3.9 to −25.0)	0.008*
ISI (Sleep)	+28.9 ± 16.7 (14.3 to 57.1)	−51.4 ± 9.3 (−8.0 to +14.3)	0.036*
Go reaction time	+8.4 ± 9.3 (−18.4 to +28.2)	−4.5 ± 20.2 (−21.7 to +17.5)	0.413
NoGo reaction time	+1.3 ± 14.5 (−6.1 to +13.6)	−4.0 ± 7.0 (−23.5 to +14.3)	0.730
AY reaction time	+1.7 ± 11.9 (−18.9 to +28.6)	−5.8 ± 7.1 (+0.8 to −16.1)	0.413
BX reaction time	−14.4 ± 17.0 (−30.3 +16.1)	+1.4 ± 7.7 (−4.7 to +13.2)	0.413
AX-CPT accuracy	+93.3 ± 13.3 (+66.7 to +100.0)	+1.6 ± 59.0 (−66.7 to +83.3)	0.032*
PBI-reaction time	+65.8 ± 19.6 (−12.3 to +500.3)	−28.9 ± 17.3 (−500.7 to +104.5)	0.90
Emotion induction	−4.9 ± 22.4 (−43.5 to +22.2)	−8.6 ± 10.3 (−26.1 to 0)	1.00
Emotion regulation Index	−6.0 ± 15.6 (−33.3 to 0)	−3.0 ± 16.2 (−25.0 to +14.3)	0.91
Head motion (absolute)	−56.7 ± 29.6 (−18.7 to −91.3)	−34.9 ± 29.0 (−5.8 to −64.0)	0.80
Head motion (relative)	−49.5 ± 27.4 (−12.5 to −77.8)	+25.7 ± 75.0 (−50.0 to +100.0)	0.40

(+) and (−) signs indicate increase and decrease, respectively, of respective outcome measure. The differences between the groups were tested using Mann–Whitney *U*-test.

^a^ Composite measure of change in clinical variables was calculated for ASRS v1.1., CGI-S, PGI-I, MADRS-S, EQ VAS.

* *p* < 0.05 is considered statistically significant.

### 3.5. fMRI and head motion

Three participants from the intervention group and two from the control group performed structural MRI, resting-state and task-related fMRI before and within one week after the end of intervention. AX-CPT was used to assess cognitive control, Go/NoGo to assess impulsive behavior, and the International Affective Picture Series (IAPS) was used to assess effortful emotion regulation with reappraisal. Preliminary analysis comparing brain activation pre- and post-intervention showed no significant changes at the group level for any of the tasks evaluated. There was, however, a greater decrease in absolute and relative head motion calculated from the fMRI experiments in the intervention group compared to the control group (Figure 2B).

#### 3.5.1. Cognitive functions

Behavioral data from the cognitive paradigms (AX-CPT, Go/NoGo and emotion regulation task, *n* = 11) are shown in Figures 2C,D and Table 3. Although inferential analysis was not within the scope of the pilot study, there were promising trends indicating that the intervention may have cognitive benefits. No such trend could, however, be discerned in the emotion induction and emotion regulation tasks (not shown).

### 3.6. Changes in self-efficacy, body awareness and physical fitness

Changes in self-efficacy (GSE-10), body awareness (BAS MQ-E) and measures of physical capacity with handgrip strength with dynamometer and balance test are shown in Table 4, where varied changes in a positive direction could be discerned in the intervention but not control group.

**Table 4:** Variables related to general health markers and fitness expressed as post-intervention % change compared to baseline for the intervention and control groups.

Outcome variables	Intervention % change ± SD (range)	Control % change ± SD (range)	*p*-value
BMI	−1.8 ± 2.0 (−4.4 to +1.3)	+0.2 ± 4.4 (−3–16 to +9.0)	0.55
Waist circumference	−1.8 ± 3.1 (−4.55 to +4.1)	−2.1 ± 1.5 (−3.7 to 0)	0.84
Heart rate	+2.3 ± 11.4 (−14.9 to +18.6)	−0.5 ± 8.3 (−12.8 to +10.5)	1.0
BT systole	−2.3 ± 10.2 (−12.3 to +14.7)	+0.5 ± 4.1 (−5.7 to +5.4)	0.57
BT diastole	−6.4 ± 14.5 (−24.0 to +16.7)	+13.8 ± 9.5 (−12.4 to +14.5)	0.41
VO2max	−1.4 ± 6.4 (−14.4 to +3.2)	+4.5 ± 7.1 (−6.4 to +14.5)	0.22
Balance	+58.5 ± 25.5 (+30.0 to +100.0)	−22.4 ± 27.9 (−15 to +57)	0.095
Hand strength right	+12.0 ± 16.9 (−6.3 to +32.3)	−1.0 ± 4.2 (−7.8 to +4.2)	0.31
Hand strength left	+8.5 ± 7.7 (0 to +20.0)	+0.36 ± 7.0 (−5.6 to +13.0)	0.15
Steps	+44.6 ± 48.4 (+6.4 to +127.5)	−3.3 ± 40.0 (−53.3 to +42.3)	0.34
SEE-SV	−58.0 ± 59 (−31.7 to +69.6)	+16.7 ± 71.8 (+3.4 to +75.0)	1.0
BAS MQ-E	−26.8 ± 15.1 (−48.9 to −5.2)	+17.0 ± 18.6 (+1.3 to +48.6)	0.016*

(+) and (−) signs indicate increase and decrease, respectively, for a given variable. The differences between the groups were tested using Mann–Whitney *U*-test.

* *p* < 0.05 is considered statistically significant.

### 3.7. Changes in general health measures and vital signs

At baseline, 81% of the randomized participants had a Body Mass Index (BMI) over 25 and were overweight and 36% had BMI >30 and were thus clinically defined as obese. Heart rate, blood pressure, VO2 extraction, BMI, and waist circumference did not noticeably differ post-intervention compared to baseline, neither in the intervention nor in the control group (Table 4).

### 3.8. Participant perception on measurements and intervention

#### 3.8.1. Perception of pre- and post-intervention measurements

All 11 participants completed a study-specific questionnaire that assessed their experience of the study. Overall, participants perceived the amount of time allocated for the pre- and post-intervention tests as reasonable and the tests as not overly strenuous and the level of difficulty was considered appropriate to the task at hand. On the other hand, participants stated that the fMRI examination and the associated cognitive tasks were mentally strenuous and took a rather long time. Overall, participant comments on the testing procedures in running text were predominantly positive regardless of whether they belonged to the intervention or control group. They commented that the tests increased their awareness of their physical and cognitive abilities and deficiencies, and that they learned about their health from the tests. One participant in the control group wrote:

*“Testing different things in this way makes me more aware of my state of health, which I think is good”*

Despite the increased awareness they said they gained from the tests, the control group reported that the tests in themselves had not affected their level of physical activity.

#### 3.8.2. Perceptions of the intervention

All participants in the intervention group who completed the study (*n* = 6) answered that they were satisfied with the content and length of the training sessions, the available time slots, and the instructors' ability to motivate them. The intervention was considered to be physically strenuous by all participants in the intervention group (6 out of 6), and to be mentally strenuous by 50% (3 out of 6) of the participants. All participants stated that the intervention contributed to improved health in general (6 out of 6) and 50% (3 out of 6) stated that the intervention, more specifically, led to a reduction in ADHD symptom in particular. Five out of 6 participants (83%) found it likely or very likely that they will continue to exercise after the end of the intervention. Participants found it easier to do the training with a group than alone by themselves. For some, the group was said to be a prerequisite for doing the exercises, as one participant wrote:

*“It has not been possible to exercise on my own at all, I have been able to go for walks. Even though I felt motivated, it didn't work”.*

Two out of 6 participants (33%) in the intervention group stated that they had trouble finding time for the exercise sessions and 4 out of 6 participants (67%) stated that they experienced some degree of negative stress trying to catch up with the sessions. For some participants, the stress decreased as the intervention progressed and as they became more used to the routine. One participant wrote:

*“In the beginning, it was hard to find time, and easy to put it off. After about halfway, it became easier to fit the training sessions into my regular schedule.”*

Participants in the intervention group expressed that participation in the intervention gave a sense of increased well-being. Four out of 6 (67%) participants described more energy and vigour, feeling happier, calmer, and more at ease with themselves as a result of the intervention.

## 4. Discussion

The primary aim of the pilot study was to investigate the feasibility and practicality of a planned randomized controlled trial (RCT) aiming to investigate the effects of a 12-week 3 times weekly mixed exercise program consisting of cardio, strength and flexibility exercises in adults with ADHD. A secondary objective was to gain experience and collect preliminary data that could help optimize the design of the planned RCT. The pilot study thus mirrored in design and logistics that of the planned RCT. The intervention was administered by experienced staff of licensed physiotherapists at the Unit for Psychiatric Physiotherapy in Region Örebro County, Sweden. Data were collected digitally using an electronic data capture platform (Smart-Trial) at baseline and 6 and 12 weeks after the start of the intervention, which will also be used in the planned RCT. Participants were recruited by advertisement in the local community from a catchment area of 300,000 with an expected population of adult ADHD patients of roughly 6,000, which lead to a steady flow of participants and a satisfactory level of retention and adherence to the study protocol. Initially, shortly after randomization, there was a sizable drop-out (20%), which however stabilized once intervention started. To encourage fidelity to protocol, a minimum participation level of 60% was set for the intervention group and participants who missed a session were offered alternative dates within the same week to make up for the missed session. Of the 11 individuals who participated in at least one post-randomization intervention session, only one participant failed to meet the 60% threshold and was thus not included in the analysis. However, even this individual, who had a participation level of 54%, attained comparable results as those with participation level of 60% or higher. Having thus noted consistent changes in the primary outcome measure as early as week 6, the cut-off level for minimum participation will be set at 50% for the planned RCT.

In the present study, there were more female than male participants in both the intervention and control groups. This, at first glance, may seem odd in light of the known gender differences in ADHD. However, gender distribution in adult ADHD has been shown to differ from that in children. Both epidemiological and clinical studies in adult ADHD have shown roughly equal prevalence in men and women (4, 6). In the planned RCT, we will thus aim for a 1:1 gender balance to reflect prevalence rates in adult ADHD.

Although this was not inferentially tested, we noted a consistently greater change from baseline in the intervention group compared to the control group for almost all outcome variables assessed, which we believe indicates that the planned RCT not only will be feasible but will also has a good chance of establishing evidence of efficacy or lack thereof for the proposed intervention. When participants were asked to rate their subjective impression of improvement (Patient Global Impression – Improvement, PGI-I), 80% in the intervention group and 0% in the control stated that they had improved “much” or “very much” (PGI-I score of “1” or “2”) at one of the two assessment periods (6 or 12 weeks). At endpoint, 40% of the intervention group and none 0% in the control group said they had improved “much” or “very much” as a result of the intervention (PGI-I score of “1” or “2”). Similar results were also seen in symptom reduction assessed using ASRS v.1.1 where 3 out of 5 participants in the intervention group but none in the control group had >25% symptom reduction. We saw similar trends in Clinical Global Impression – Severity (CGI-S), quality of life assessed using the EQ VAS, body awareness assessed with BAS-MQ and sleep quality and quantity assessed with ISI. A composite score of “improvement” calculated as percentage change from baseline for 5 clinical outcome variables (ASRS v.1.1, CGI-S, PGI-I, MADRS-S and EQ VAS) showed a significant trend towards a better outcome in the intervention group compared to the control group. These preliminary trends are compatible with studies reporting that regular physical exercise improves symptoms and cognitive functioning in children and adolescents with ADHD (28, 29, 32). Also, a meta-analysis that evaluated the efficacy of different types of exercise programs for children with ADHD showed that mixed exercise programs of the type used in the present study had advantages over other exercise programs (31), which further strengthens our preliminary findings.

Regarding brain imaging, our target level in the pilot study was that 50% of participants would undergo MRI investigation. Three participants from the intervention group and 2 from the control group completed the structural, resting-state and task-related fMRI investigations before and within one week after completing intervention. Overall, participants stated that the MRI investigations and the associated cognitive paradigms were rather cumbersome and took a long time to complete, and one participant who did baseline MRI declined to do post-intervention MRI. The target level for the planned RCT is to have a minimum of 25 participants from the control and intervention groups to undergo MRI investigations before and within one week after the end of the intervention. Jiang et al. (2022) using resting state fMRI reported that aerobic exercise improved executive function with concomitant changes in spontaneous activations in the left middle and right superior frontal gyri (30), two prefrontal cortex areas widely known to be important for attention and top-down cognitive control (56, 57). Because of the small sample size in the present study, the fMRI data were not further analyzed at the group level. However, we noted that head movement during the MRI investigations showed trends indicating that it could be a useful surrogate measure of hyperactivity, something that has also been shown in earlier studies (58, 59). In the adequately powered planned RCT, it will also be interesting to see if the structural differences in certain brain regions between healthy controls and ADHD patients that have been reported in some studies (60) could be modified by the mixed exercise program.

The results from the study-specific questionnaire showed that overall participants in the intervention group were very satisfied with what they had achieved at the end of the study. However, some participants also expressed difficulties with planning and organization, both in the questionnaire they completed and spontaneously during intervention sessions. To find time for physical exercise and to manage the negative stress that this caused in their daily life were reported as major hinders for adherence. Given the impairment in cognitive functioning people with ADHD have, difficulties with planning and organization are expected to be obstacles to achieving sustained participation in the planned 12-week RCT. We have, therefore, decided to add a third intervention arm to the planned RCT in which participants, besides the mixed exercise program, will also receive cognitive intervention by an occupational therapist with the aim of improving planning, organization and time management skills. To evaluate potential benefits of this third arm, three assessment instruments will be added. Assessment of Time Management Skills (ATMS-S) to evaluate time management, planning, organization and emotional regulation in relation to time (61), Adult ADHD Quality of Life questionnaire (AAQoL) to evaluate overall daily functioning (62) and Satisfaction with daily occupations and occupational balance questionnaire (SDO-OB) (63, 64) to evaluate satisfaction with daily activities and perceived balance between activities.

Because of the nature of the intervention, an unsurmountable problem in the design of the present study was the expectation bias created by the fact that participants could not be blinded as to the type of intervention they received, neither in the intervention group nor in the control group. One consequence of this was that participants during clinician-rated assessments may inadvertently mention the type of intervention they received and compromise the integrity of evaluator blinding. We see this as a major weakness of both the pilot study and the planned RCT, which might lead to an overestimation of the effect of the intervention. To mitigate this, participants will be instructed to not reveal directly or indirectly as to which group they belonged to. In this regard, we also hope that participant- and rater-independent objective outcome measures such as structural and function MRI and measures of bodily function (heart rate, blood pressure, etc.), after adequate blinding during analysis, would to some extent counterbalance this weakness. An example of an objective measure that could be mentioned here is head motion, which is measured with great precision during fMRI, which in previous studies has been shown to correlate well with inattention and hyperactivity (58, 59). In the pilot study, out of the 5 participants who did pre- and post-intervention fMRI, 3 of 3 participants from the intervention group had a 15% decrease in head motion at the end of the intervention compared to baseline, while no such reduction was observed in the 2 participants from the control group. Other participant and rater independent objective outcome measures are reaction time and error rate assessed during the cognitive control paradigms (AX-CPT and Go No/Go), which could also contribute with unbiased measures of some effects of the intervention.

Of the 14 enrolled participants, 3 dropped out before participating in any session of the intervention. The reason for drop-out was mainly reported as lack of time. Taking this and the result of the pilot study into consideration, we intend to do the following modifications in the planned RCT. To reduce drop-out and improve adherence, we plan to add a third arm to the planned RCT where participants will receive physical exercise together with cognitive intervention to improve time-management skills. We will thus investigate the effect of two active interventions, i.e., physical exercise with and without cognitive intervention compared to a control group receiving standard care only. A second modification to the planned RCT that we plan to do is to increase the sample size from the previously planned 75 participants to 120 participants to accommodate the third arm of the study. To this end, we plan to apply for additional ethical approval as our current approval has a sample size of only 75 participants. Another adjustment to the planned RCT is to include clinician-rated Clinical Global Impression – Improvement (CGI-I) as a complement to the participant-rated Global Impression – Improvement (PGI-I).

## 5. Conclusion

The present pilot study showed encouraging results that indicate that the planned RCT is indeed feasible, with an expected steady flow of included participants and satisfactory levels of retention and adherence. The results from the pilot study are also indicative that the intervention has fair odds of being effective in improving both core ADHD symptoms and general physical and psychiatric health outcome measures. Furthermore, the modifications that are being made in the planned RCT, based on the experiences gained in the pilot study, are expected to improve and optimize the design of the planned RCT study.

## Data Availability

The raw data supporting the conclusions of this article will be made available by the authors, without undue reservation.
